# Remote monitoring of building oscillation modes by means of real-time Mid Infrared Digital Holography

**DOI:** 10.1038/srep23688

**Published:** 2016-04-01

**Authors:** Pasquale Poggi, Massimiliano Locatelli, Eugenio Pugliese, Dario Delle Donne, Giorgio Lacanna, Riccardo Meucci, Maurizio Ripepe

**Affiliations:** 1 Istituto Nazionale di Ottica-CNR, Firenze, Italy; 2 Dipartimento di Scienze della Terra, Università degli Studi di Firenze, Firenze, Italy; 3 Dipartimento di Scienze della Terra e del Mare, Università degli Studi di Palermo, Palermo, Italy

## Abstract

Non-destructive measurements of deformations are a quite common application of holography but due to the intrinsic limits in the interferometric technique, those are generally confined only to small targets and in controlled environment. Here we present an advanced technique, based on Mid Infrared Digital Holography (MIR DH), which works in outdoor conditions and provides remote and real-time information on the oscillation modes of large engineering structures. Thanks to the long wavelength of the laser radiation, large areas of buildings can be simultaneously mapped with sub-micrometric resolution in terms of their amplitude and frequency oscillation modes providing all the modal parameters vital for all the correct prevention strategies when the functionality and the health status of the structures have to be evaluated. The existing experimental techniques used to evaluate the fundamental modes of a structure are based either on seismometric sensors or on Ground-based Synthetic Aperture Radar (GbSAR). Such devices have both serious drawbacks, which prevent their application at a large scale or in the short term. We here demonstrate that the MIR DH based technique can fully overcome these limitations and has the potential to represent a breakthrough advance in the field of dynamic characterization of large structures.

The dynamic characterization of an engineering structure implies the knowledge of the fundamental oscillation modes and has direct implications in evaluating the health status and the correct vulnerability of the structure^1^. In fact, it discloses structural deficiencies and provides important information on the elastic response of the edifice to seismic forcing, environmental deterioration (wind, rain and natural calamities in general) or anthropogenic activities (rail or vehicular traffic, underground works, etc.).

The dynamic response of the structure is defined by its geometry and by the physical properties of the construction materials. Starting from these features it is possible to model the three-dimensional behaviour of the building to the external forcing also in non-linear rheology conditions. This approach is extremely sophisticated but also time-demanding and then of limited application when large and quick response on the vulnerability in large areas is required.

Currently, a precise dynamic characterization, but limited to the elastic field, can be obtained by the use of seismometers^2^. The technique consists in positioning several seismic sensors inside the building and recording the displacements induced by ambient noise along the three spatial directions. If a sufficiently high number of seismometers is employed, it is possible to obtain the modal parameters of the building (resonance frequency, damping and modal shape) using Frequency Domain Decomposition (FDD)^3^. The experimental technique is faster than 3D modelling and it provides very accurate and sensitive measurements, up to 0.1 *μm* of amplitude oscillations, nevertheless it has some critical drawbacks. Indeed, the positioning of seismic detectors inside the structure usually requires complicated and time-consuming bureaucratic procedures, especially in presence of architectural or historical constraints or in case of damaged structures or when the operational activity of the building (e.g. hospitals) cannot be stopped.

A recent alternative technique to evaluate building oscillation modes is represented by Ground-based Synthetic Aperture Radar (GbSAR)^4^. Differently from the seismometric technique, the use of radar is remote and does not require entering into the structure to be investigated. This technique, however, has a displacement resolution of about 0.1 *mm* much lower than the typical micrometric or sub-micrometric oscillations of standard buildings and therefore its employment is restricted mainly to wide oscillating structures like bridges or towers. Moreover, with the radar technique there is a large uncertainty regarding which part of the structure is generating the signal. For this reason quite often reflecting targets are placed on the building to be sure on the position of the oscillations. The SAR resolution is nearly comparable to that of commercially available laser devices, employing the time of flight or other techniques, which is about 1 *mm *5.

We here propose a method which overcomes the critical issues related to the standard techniques, applying for the first time a technique based on MIR DH^6^ to retrieve oscillation modes of large edifices. Digital Holography (DH) is an interferometric imaging technique^7^, directly derived from classical holography^8^, providing amplitude and phase information on the wavefront scattered by an object irradiated with coherent radiation. Holograms result from the interference between the radiation scattered by the target (the so-called object beam, OB) and a reference beam (RB) directly impinging on a digital recording device (see Methods).

In the so-called off-axis configuration, the reference and the object beams are sufficiently inclined between each other by an angle *θ* that, during hologram reconstruction, the two diffraction orders are completely separated and a clear reconstructed image is obtained. The maximum admitted value of the angle *θ* is limited by the Shannon sampling theorem due to the finite dimension of the camera pixels. This is a specific limit of DH with respect to classical holography which significantly limits its field of view. Moreover, in general, DH is strongly perturbed by environmental vibrations as any interferometric technique. The above constraints are particularly restrictive in the visible range. Indeed, despite whole-field non-contact measurements of vibrating objects has been successfully demonstrated by means of visible DH in combination with high speed CCD camera acquisition^9^, this region of the electromagnetic spectrum is not well suited for open space measurements of micrometric oscillations of large size structures. On the contrary, infrared DH inherently benefits from wavelength scaling of both mechanical stability requirements and linear field of view^10^. In fact, the fringe spacing for a certain value of *θ* increases with the wavelength so that the requirements of the Shannon sampling theorem become less restrictive and a proportionally larger field of view is obtained.

Recent developments of MIR DH based on micro-bolometric arrays and *CO*_2_ lasers^11^ or, more recently, MIR Quantum Cascade Lasers^12^, demonstrate the possibility to record holographic videos of large dynamic scenes even outside a laboratory framework and have been used to detect human targets beyond a curtain of smoke and flames^13^.

At the same time, several innovative results have been obtained in the field of vibration detection in non destructive tests employing infrared interferometric techniques closely related to digital holography as speckle interferometry. In particular, this technique has been used in vibration analysis of small fast moving objects^14^, in non destructive tests in aeronautics^15^ and in thermal imaging^16^.

In the following we show that MIR DH can provide remote real-time deformation displacements of large structures with a resolution comparable to seismometric methods. Furthermore, MIR DH allows clear non-contact imaging of the monitored area offering a complete displacement map of the investigated structure.

## Results and Discussion

**Figure 1:**
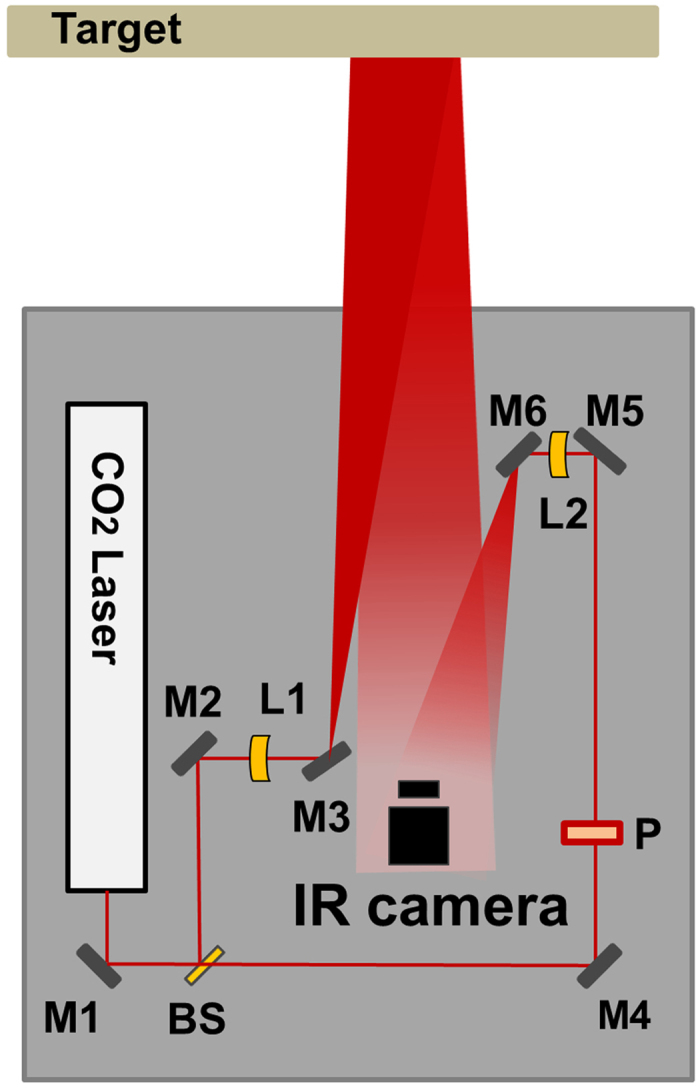
Experimental set-up. M1, M2, M3, M4, M5, M6 mirrors, BS 90/10 ZnSe beam splitter, P Polarizer, L1, L2 ZnSe lenses.

We demonstrate that MIR DH can be used in open field to detect the oscillation modes of large size structures, thus opening a new use of this technique. To this purpose, we developed a compact MIR DH set-up (see Fig. 1 and Methods Section for a detailed description) employing a microbolometric camera with 640 × 480 pixels and a space-saving radiofrequency *CO*_2_ laser with an output power 60 *W* CW at 10.6 *μm* and a coherence length longer than 50 *m*. Such a portable set-up has been used in open space and without damping systems to test its capability for micrometric displacement measurements.

The apparatus is able to record sequences of IR digital holograms from which it is possible to extract amplitude and phase images of the wavefront back-scattered from the investigated target. Each pixel of the reconstructed images represents a certain area *A* of the structure, whose dimensions, *A*_*x*_ and *A*_*y*_, are determined by the intrinsic resolution formula of digital holography^17^


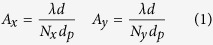


where *λ* is the wavelength, *d* is the distance between target and detector, *N*_*x*_ and *N*_*y*_ are the pixel numbers along each direction and *d*_*p*_ is the detector pixel pitch. The holographic amplitude images are employed to visualize the target and to select a particular zone of interest while the phase images allow to retrieve the micrometric displacements of the investigated area.

**Figure 2:**
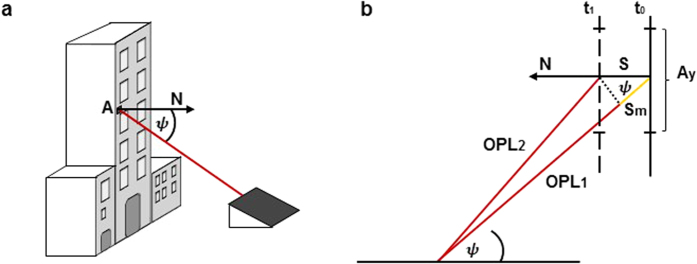
Optical path length variation and surface displacement. (**a**) Irradiation direction (red) with respect to the normal direction *N* to the surface of the building. (**b**) Optical path length (OPL) variation *S*_*m*_ (yellow) and surface displacement *S* along the direction *N* occurring between time *t*_0_ and *t*_1_.

The distance between the laser output and the IR detector (see Fig. 1) can be considered negligible with respect to the distance between the target and the IR detector so that, in first approximation, the laser beam direction and the detection direction can be considered spatially coincident. With this assumption, the oscillation amplitude of the building can be recovered directly from the optical path length variation of the OB. Indeed the radiation impinging on each area A of the structure undergoes an optical path length variation 2*S*_*m*_ which is related to the displacement *S* of the area A along to the normal direction N to the surface itself, (Fig. 2(a,b)) according to


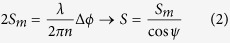


where Δ*ϕ* is the observed phase variation due to the target displacement, *ψ* is the angle between the OB direction and the normal *N* to the surface and *n* is the air refractive index at *λ* = 10.6 *μm*. The displacement of the irradiated area *A* determines a phase variation associated with the pixel representing the area *A* and it is connected to the length *S*_*m*_ as shown in Fig. 2(b).

The temporal evolution of the phase variation Δ*ϕ* (or, equivalently, of the displacement *S*) is obtained as the algebraic sum of phase variation values over consecutive frames of the holographic sequence (see Methods). In general, this signal contains frequency contributions coming from the structure oscillation modes as well as from the experimental set-up vibrations and, therefore, it must be filtered by means of an appropriate band pass filter in order to eliminate the unwanted contributions. The main spurious frequency component (around 9 *Hz*) arises in the set-up (see Methods) due to the laser integrated air cooling system.

**Figure 3:**
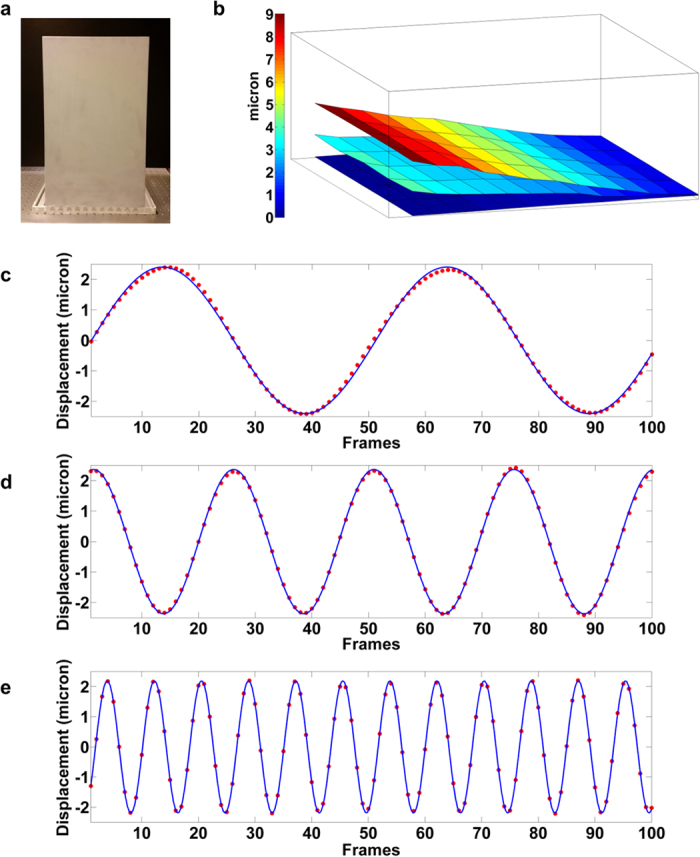
Holographic reconstruction of the aluminium plate oscillation. (**a**) The aluminium plate. (**b**) 3D reconstructions of the aluminium plate at three different displacement values. (**c**–**e**) Holographic reconstruction of the driven signal at 0.5, 1 and 3 *Hz*, respectively. The experimental data (red points) are fitted using a sinusoidal function (blue curve) to determine amplitude and frequency of the oscillation.

Preliminary laboratory tests were performed on a moving aluminium plate (30 *cm* × 50 *cm* × 0.5 *cm*) forced by a known periodic signal (see Fig. 3(a)). The target was fixed on the table and positioned 7 *m* away from the emission/detection apparatus. A piezoelectric translator was fixed to the back upper side of the plate. Three sinusoidal signals, with the same amplitude (460 *V*) and different frequencies (0.5 *Hz*, 1 *Hz* and 3 *Hz*) were applied to the piezoelectric translator. In this condition the plate was estimated to oscillate with a maximum amplitude of 9 ± 1 *μm* at its top edge.

The sinusoidal displacement of the plate at half of its height was accurately reconstructed starting from the holographic phase images (Fig. 3(c–e)). The experimental holographic data (red points in Fig. 3(c–e)), were fitted by using a sinusoidal function to determine amplitude and frequency of the oscillation. The best curve has been calculated using the method of nonlinear least squares employing the Trust Region Algorithm^18^. The R-square goodness of fit has been used to check the agreement with the experimental data resulting in *R*^2^ = 0.999. The estimated values of amplitudes and frequencies reproduce the expected ones within the experimental errors (*RMSE* ≅ 0.1 *μm*).

The 3D motion of the aluminium plate reconstructed by successive displacements is reported in Fig. 3 at three different times. In order to reduce the noise in each reconstructed phase image, the target was divided into smaller areas and the mean displacement over each area was calculated.

**Figure 4:**
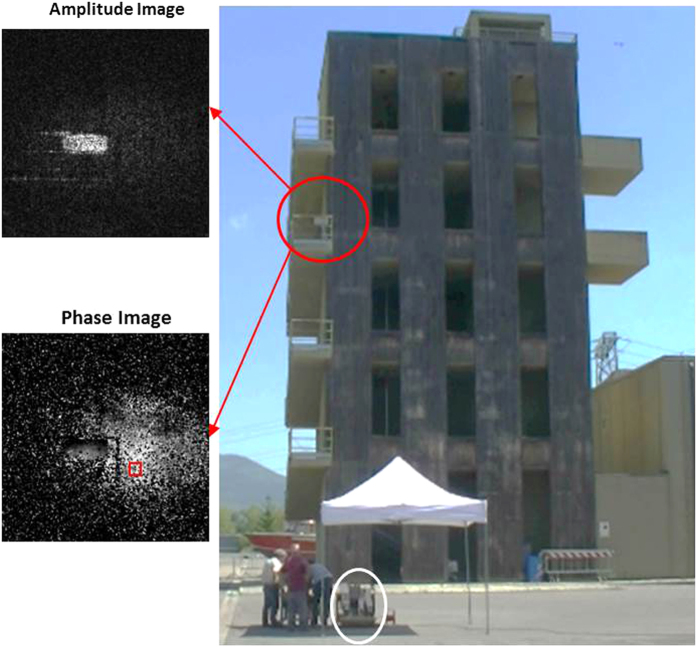
Real condition measurement campaign. The training tower of the Florence Firefighters Department; investigated area (red circle) and the corresponding amplitude and phase images on the left. The red square indicates the set of pixels from which the elaborated signal was extracted. The measurement apparatus is shown inside the white circle.

After these preliminary tests, holographic measurements have been carried out in a real case by monitoring the training structure of the Firefighters Department in Florence (Fig. 4) and compared with seismometric data. The investigated structure is a typical 4-story reinforced concrete building with a rectangular base of about 10 *m* × 7 *m* large.

Two seismic stations were positioned in the building, one at the base and the second at the third floor on the same vertical axis. Both stations were equipped with a 3 component seismometer (Lennartz 3D/5s), the seismic data being digitized using a 24 bit Guralp CMG24 Digitizer at 100 *Hz*. The time synchronization between stations is achieved by means of the Global Positioning System (GPS) signal. The seismic detectors were oriented in order to collect displacements in the horizontal plane along the direction *N*, normal to the main facade of the structure.

The MIR DH measurement apparatus was positioned at a distance of 14 *m* from the structure and oriented in order to irradiate the area nearby the third floor seismometer with an angle *ψ* = 38° (see Figs 2(a) and 4). In Fig. 4 we show a typical amplitude and phase reconstruction of the laser irradiated surface. According to the resolution formula, the reconstruction pixel pitch dimensions at this recording distance (about 18 *m*) are about 12 *mm* × 16 *mm* which represent an estimation of the measurement precision.

**Figure 5:**
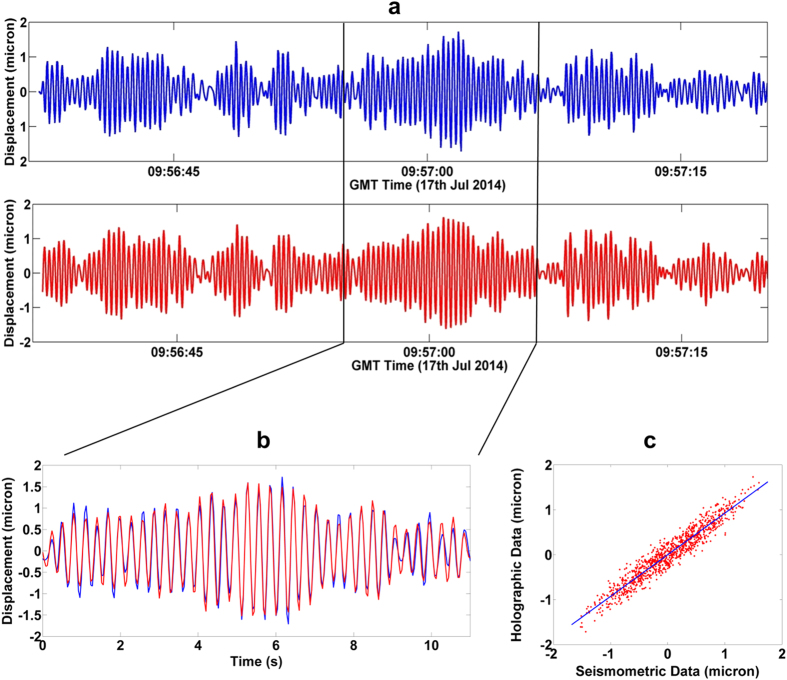
Holographic data vs seismometric data. (**a**) Displacement recovered with the holographic method (blue line) and with the seismometric method (red line) in a 45 *s* acquisition. (**b**) Comparison between holographic signal (blue line) and seismometric (red line) signal. (**c**) Correlation between holographic and seismometric data.

The MIR DH acquisitions were synchronised using the GPS signal too. Several acquisitions (45 *s* long) were collected, in different periods of the day and choosing different portions of the irradiated surface. For each acquisition, the time evolution of the displacement was measured from the holographic phase images, after filtering and the correction of the amplitude along the line of sight for the horizontal displacement by the factor cos (38°). The comparison between the holographic and seismometric signal is shown in Fig. 5(a,b). The high degree of correlation between the two techniques is displayed in Fig. 5(c) with an *RMSE* ≅ 0.2 *μm*. The frequency of the Firefighters building oscillation first mode (3.4 *Hz*) is correctly recovered by the MIR DH (see Fig. 5(b)), while the holographic oscillation amplitudes agree with the seismometric ones within a resolution of 0.1 *μm*. It is important to note that in order to detect a vectorial displacement of the structure it is necessary to move the experimental system in front of the other facades of the structure and monitor the corresponding displacements.

## Conclusions

In this paper, we show how the MIR DH technique is able to work in the field and to measure in real-time the oscillation modes of large size structures. This technique has a resolution of about 0.1 *μm*, fully comparable with that of standard seismometers but with the remarkable advantage of being a remote and thus contactless technique like the radar.

In particular, it provides static deflection measurements (e.g. structural loading test), real-time monitoring of slowly occurring displacements (e.g. collapse hazards) and vibration measurements (e.g. modal analysis).

The developed MIR DH technique offers the possibility to simultaneously monitor the behaviour of a large portion of a structure without having to enter inside the buildings (like for the seismic) or to fix reflecting targets on its surface (like for the radar). This largely opens the application field of MIR DH technique to structural engineering, seismic vulnerability and cultural heritage preservation. Our technique is also well suited for the non-invasive monitoring of the environmental deterioration and the aging process of historical monuments and artistic heritage.

We suggest that MIR DH has the features requested to characterize at a large scale the elastic behaviour of engineering structures and it represents a fundamental breakthrough when vulnerability of urban infrastructures has to be assessed in not accessible or hazardous area.

## Methods

### Set-up

The experimental set-up is shown in Fig. 3(b). It includes a radiofrequency pumped *CO*_2_ laser (by Universal Laser Systems), emitting 60 *W* CW of linearly polarized radiation at 10.6 *μm* wavelength and a room-temperature microbolometric thermocamera (Miricle 307 k by Thermoteknix). The focal plane array is composed of 640 × 480 amorphous Si elements with 25 *μm* × 25 *μm* pixel pitch. The camera operates at a frame rate of 25 *Hz*. The laser beam is divided by means of a beam splitter reflecting 90% of the radiation in the Object Beam (OB) and transmitting the remaining 10% in the Reference Beam (RB). The OB is expanded by means of a diverging ZnSe lens and it is directed toward the area to be investigated; the RB is re-directed, by means of gold coated mirrors, toward a diverging ZnSe lens before impinging on the thermocamera (used without objective). A polarizer, inserted in the RB path, balances the intensity of the reference beam with respect to the intensity of the scattered radiation in order to optimize the visibility of the interferometric pattern. The set-up is mounted on a 60 *cm* × 90 *cm* breadboard and fixed to an inclined frame. The collected interferograms are elaborated in real-time as described in the following section.

### Digital Holography recording and real-time displacement reconstruction

In Digital Holography, the interference between the radiation coming from the object, the so-called Object Beam (OB), and the reference radiation, Reference Beam (RB), is recorded on a digital recording device and constitutes the hologram H. We can write H as follows


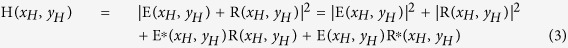


where E, E^∗^, R, R^∗^ are the complex and complex conjugate amplitudes of the object and reference beam, respectively; *x*_*H*_, *y*_*H*_ are the coordinates on the hologram plane.

When the product R(*x*_*H*_, *y*_*H*_)H(*x*_*H*_, *y*_*H*_) is considered, the real and imaginary contributions of the OB wavefront emerge as follows


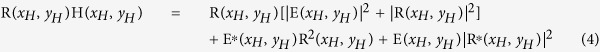


The holographic reconstruction of the OB wavefront is obtained by means of a numerical implementation of the Rayleigh-Sommerfeld diffraction integral^19^ reproducing the diffraction effect operated by the optical transmittance constituting the hologram


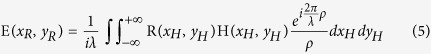


where R(*x*_*H*_, *y*_*H*_) is a numerical replica of the RB, *x*_*R*_, *y*_*R*_ are the coordinates on the reconstruction plane; *ρ* is the distance between the generic point (*x*_*H*_, *y*_*H*_) and the generic point (*x*_*R*_, *y*_*R*_), *λ* is the wavelength of the employed radiation. The intensity I(*x*_*R*_, *y*_*R*_) and the phase *ϕ*(*x*_*R*_, *y*_*R*_) of the object wavefront on the reconstruction plane are calculated from


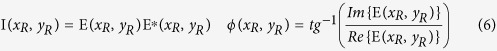


For small values of *x*_*H*_, *y*_*H*_, *x*_*R*_, *y*_*R*_ compared with *ρ* it is possible to adopt the Fresnel approximation. In this approximation, the Rayleigh-Sommerfeld integral becomes the so-called *Fresnel transformation*. Due to its mathematical form the Fresnel transformation represents the Fourier transform of the function 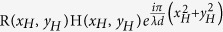



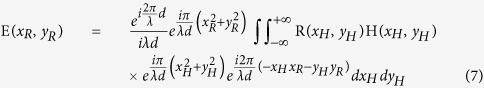


The holographic pattern H is digitized into a 2D M × N matrix and the previous integral turns into a Discrete Fourier Transform (DFT). In such a way, the complex amplitude of the object beam becomes a discrete function^7^


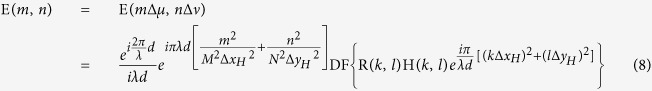


where the reconstructed pixel dimensions Δ*μ*, Δ*ν* are connected to the hologram pixel dimensions Δ*x*_*H*_, Δ*y*_*H*_ and the indices *m*, *n*, *k*, *l* run from 0 to *M*, *N*.

As E(*m*, *n*) represents the complex amplitude of the reconstructed object beam corresponding to the pixel (*m*, *n*), it is possible to rewrite it as E(*m*, *n*) = |E(*m*, *n*)|*e*^*iϕ*(*m*,*n*)^.

A dedicated Matlab routine efficiently computes the above DFT by means of an FFT algorithm returning the amplitude and phase of the object wavefront. Since the holograms are acquired at the maximum frame rate of 25 *Hz*, the phase difference between two successive holograms Δ*ϕ*_*k*,*k*−1_(*m*, *n*) = *ϕ*(*m*, *n*; *t*_*k*_) − *ϕ*(*m*, *n*; *t*_*k*−1_) corresponds to the phase variation in the time interval Δ*t*_*k*,*k*−1_ = *t*_*k*_ − *t*_*k*−1_ = 0.04 *s*. The maximum frame rate of the camera imposes the maximum oscillation mode frequency which is possible to detect according to the Shannon sampling theorem.

The macroscopic displacement *S* is evaluated by the following expression


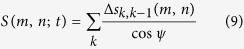


where the displacements Δ*s*_*k*,*k*−1_(*m*, *n*) are obtained from 
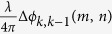
, cos *ψ* is the angular correction factor and 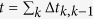 is the discretized time. The power spectrum of the displacement *S*(*m*, *n*; *t*) contains spurious frequency components associated with the set-up vibrations. In order to eliminate such spurious components, the output signal is thus processed by means of a Butterworth band pass filter of the fourth order with a frequency window centred on the value of interest. The signal-noise ratio can be enhanced performing the mean over a small number of pixels (i.e. 5 × 5 submatrix) representative of a small region of the examined surface.

Our Matlab routine includes the following algorithms improving the quality of the reconstructed images: i) suppression of the zero diffraction order by means of one of three different numerical filters applied to the acquired holograms (subtraction of the average intensity value, high pass filter, band pass filter); ii) speckle noise reduction; iii) hologram processing with a Gaussian apodization mask, aimed to reduce the low frequency noise arising from Fourier transformations due to the finite detector dimensions; iv) zero padding procedure^20^ improving the final image resolution.

## Additional Information

**How to cite this article**: Poggi, P. *et al*. Remote monitoring of building oscillation modes by means of real-time Mid Infrared Digital Holography. *Sci. Rep*. **6**, 23688; doi: 10.1038/srep23688 (2016).

## References

[b1] Hearn G. & Testa R. B. Modal analysis for damage detection in structures. Journal of Structural Engineering 117, 3042–3063 (1991).

[b2] Brownjohn J. M. W. Ambient vibration studies for system identification of tall buildings. Earthquake Engineering Structural Dynamics 32, 71–95 (2003).

[b3] Brincker R., Zhang L. & Andersen P. Modal identification of output-only systems using frequency domain decomposition. Smart Materials and Structures 10, 441–445 (2001).

[b4] Pieraccini M., Papi F. & Rocchio S. Interferometric RotoSAR. Electron. Lett. 51, 1451–1453 (2015).

[b5] Berkovic G. & Shafir E. Optical methods for distance and displacement measurements. Adv. Opt. Photon. 4, 441–471 (2012).

[b6] Allaria E. . Digital holography at 10.6 *μm*. Optics Comm. 215, 257–262 (2003).

[b7] Schnars U. & Jueptner W. Digital holography. Digital Hologram Recording, Numerical Reconstruction, and Related Techniques. (Springer, Germany, 2005).

[b8] Gabor D. A new microscopic principle. Nature 161, 777 (1948). 18860291 10.1038/161777a0

[b9] Fu Y., Pedrini G. & Osten W. Vibration measurement by temporal Fourier analyses of a digital hologram sequence. Appl. Opt. 46, 5719–5727 (2007). 17694119 10.1364/ao.46.005719

[b10] Georges M. P. . Digital holographic interferometry with *CO*_2_ lasers and diffuse illumination applied to large space reflector metrology. Applied Optics 52, A102 (2013). 23292383 10.1364/AO.52.00A102

[b11] Paturzo M. . Optical reconstruction of digital holograms recorded at 10.6 μm: route for 3D imaging at long infrared wavelengths. Opt. Lett. 35, 2112–2114 (2010). 20548403 10.1364/ol.35.002112

[b12] Ravaro M. . Mid-infrared digital holography and holographic interferometry with a tunable quantum cascade laser. Opt. Lett. 39, 4843–4846 (2014). 25121889 10.1364/OL.39.004843

[b13] Locatelli M. . Imaging live humans through smoke and flames using far-infrared digital holography. Opt. Express 21, 5379–5390 (2013). 23482109 10.1364/OE.21.005379

[b14] Vandenrijt J. F., Thizy C. & Georges M. P. Vibration analysis by speckle interferometry with CO_2_ lasers and microbolometers arrays. *Imaging and Applied Optics 2014, Optical Society of America-OSA Technical Digest (online)* DTh4B.8 (2014).

[b15] Vandenrijt J. F. . Mobile speckle interferometer in the long-wave infrared for aeronautical nondestructive testing in field conditions. Opt. Eng. 52(10), 101903 (2013).

[b16] Georges M. P. Speckle interferometry in the long-wave infrared for combining holography and thermography in a single sensor applications to nondestructive testing: the fantom project. Proc. SPIE 9525, 95251L (2015).

[b17] Picart P. & Leval J. General theoretical formulation of image formation in digital fresnel holography. J. Opt. Soc. Am. 25, 1744–1761 (2008). 10.1364/josaa.25.001744 18594633

[b18] Björck A. Numerical methods for least squares problems. (SIAM, Philadelphia, 1996).

[b19] Born M. & Wolf E. Principles of optics, Electromagnetic theory of propagation, interference and diffraction of light. (Cambridge University Press, UK, 1999).

[b20] Ferraro P. . Recovering image resolution in reconstructing digital off-axis holograms by fresnel-transform method. Appl. Phys. Lett. 85, 2709–2711 (2004).

